# Extremely Rare Interbreeding Events Can Explain Neanderthal DNA in Living Humans

**DOI:** 10.1371/journal.pone.0047076

**Published:** 2012-10-24

**Authors:** Armando G. M. Neves, Maurizio Serva

**Affiliations:** 1 Departamento de Matemática, Universidade Federal de Minas Gerais, Belo Horizonte, Minas Gerais, Brazil; 2 Dipartimento di Ingegneria e Scienze dell’Informazione e Matematica, Università dell’Aquila, L’Aquila, Italy; University of Florence, Italy

## Abstract

Considering the recent experimental discovery of Green *et al* that present-day non-Africans have 1 to 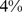 of their nuclear DNA of Neanderthal origin, we propose here a model which is able to quantify the genetic interbreeding between two subpopulations with equal fitness, living in the same geographic region. The model consists of a solvable system of deterministic ordinary differential equations containing as a stochastic ingredient a realization of the neutral Wright-Fisher process. By simulating the stochastic part of the model we are able to apply it to the interbreeding ofthe African ancestors of Eurasians and Middle Eastern Neanderthal subpopulations and estimate the only parameter of the model, which is the number of individuals per generation exchanged between subpopulations. Our results indicate that the amount of Neanderthal DNA in living non-Africans can be explained with maximum probability by the exchange of a single pair of individuals between the subpopulations at each 77 generations, but larger exchange frequencies are also allowed with sizeable probability. The results are compatible with a long coexistence time of 130,000 years, a total interbreeding population of order 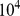 individuals, and with all living humans being descendants of Africans both for mitochondrial DNA and Y chromosome.

## Introduction

The question of whether all of us, living humans, descend exclusively from a recent (i.e. 100,000–200,000 years old) anatomically modern African population which completely replaced archaic populations in other continents, or if Africans could have interbred with these local hominids has been the subject of a long lasting and interesting debate. The first of these possibilities, known as Out of Africa model, is based mainly on genetic evidence [1] further supported by palaeontological [2] and archaeological findings [3]. The latter, known as Multiregional model, on the contrary, has been more supported by morphological studies [4], but recently it has also been found consistent with genetic data [5]. A third, intermediate possibility, known as assimilation model [6], suggests that Africans may have interbred with local archaic hominids to a limited extent.

The decision of which model correctly describes the origin of *Homo sapiens* is obscured by the intricacies of the statistical methods proposed for evaluating the models themselves. Examples of such intricate methods, their conflicting conclusions and subsequent debate are given in [5]–[7].

Until recently, the majority of the scientific community seemed to favour the Out of Africa model, but things changed radically in 2010 with a paper by Green *et al* [8]. Among other important achievements, this seminal paper provided the first direct evidence of interbreeding of modern humans with archaic hominids, Neanderthals in this case. By direct evidence we mean having sequenced Neanderthal nuclear DNA and showing that this DNA is more similar to nuclear DNA of living non-Africans than to nuclear DNA of living Africans.

Based on their findings that all non-Africans have a similar proportion of Neanderthal genes, and also on archaeological evidence [9], Green *et* *al* suggested [8] that interbreeding between anatomically modern Africans and Neanderthals might have occurred in the Middle East before expansion of Africans into Eurasia, at a time in which both subpopulations coexisted there. According to Bar-Yosef [10], the Skhul and Kafzeh caves in Israel have been occupied both by anatomically modern Africans and by Neanderthals, changing hands between one group and the other several times over a period of more than 130,000 years. Although we do not know exactly when, where, how and for how long the two groups interacted, it seems reasonable that some interaction did occur in the Middle East and that it may have lasted for a long time. This is the hypothesis we assume in this paper.

We will describe by a simple and realistic model the dynamics of two *subpopulations* – African ancestors of Eurasians (AAE, for short) and Middle Eastern Neanderthals (MEN) – interbreeding at a slow rate. For simplicity sake, we will suppose that the total population is fixed, with a prescribed stochastic mechanism for fluctuation in subpopulation sizes, and that all individuals have the same fitness, regardless of the subpopulation they belong to. With these assumptions, the model will turn out to contain a single parameter – the rate of exchange 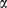 of individuals between the AAE and MEN subpopulations. We will find the probability density for this parameter by requiring that the MEN will be extinguished and present day non-African humans have between 1 and 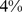 nuclear DNA of Neanderthal origin, according to the data of Green *et al* [8]. The result of having 1 to 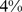 of Neanderthal DNA in present day non-Africans is attained with maximum probability for 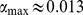, which amounts to one pair of individuals being exchanged between the two subpopulations every 77 generations. The mean value of 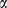 is 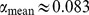, which corresponds to one pair of individuals exchanged at each 12 generations. Moreover, our results are compatible with all present day humans being descendants of Africans both for mitochondrial DNA (mtDNA) and Y chromosome, total population size of order 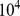 individuals, and interaction between subpopulations lasting 130,000 years.

Our work should be compared with the results of a recent paper [11] by Currat and Excoffier. Although the model used in [11] is rather different of ours, both works agree in the conclusion that the interbreeding between African anatomically modern humans and Neanderthals must have been small in order that the experimentally observed [8] introgression rate of 1 to 4% of Neanderthals into non-Africans be produced. Whereas Currat and Excoffier [11] state that the interbreeding success rate between humans and Neanderthals must have been below 2%, we prefer thinking of a small interbreeding rate between the two groups, perhaps due to cultural differences. The two models produce different outcomes, and in the Discussion section we propose experimental tests which may discriminate between them.

We would like to point out that the present research is coherent with our results [12]–[17] previous to the 2010 discovery [8]. In those works, contrary to the mainstream interpretation of mtDNA data at that time, we underlined the possibility of mating between Neanderthals and Africans.

As there seems to be no sign of Denisovan [18] contribution to the genes of most living Eurasians [19], [20], including Europeans and Han Chinese, we are justified in not considering in this paper admixture of Africans with Denisovans. As more experimental results may justify it, or not, extension of our results to interbreeding also with Denisovans may be considered.

## Analysis

### The model

Consider a population of constant size equal to 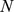 individuals, divided into two subpopulations labeled 1 and 2. Suppose also that generations are non-overlapping, that the number of generations is counted from past to future, and that reproduction is sexual and individuals are diploid. Half of each subpopulation will be of male individuals and the other half will consist of females. Subpopulation 1 will be associated with the AAE and subpopulation 2 with MEN.

We also suppose that the subpopulations had lived isolated from each other for a long time before they met. We will start generations count at the instant 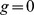 in which subppulations met. Although similar enough to permit reproduction between individuals belonging to different subpopulations, the isolation time is supposed to be large enough so that in some *loci* alleles exclusive to each subpopulation appeared. More exactly, we assume that, besides a majority of alleles common to both subpopulations, there exists also a large set of *loci* occupied by alleles which were exclusive of subpopulation 1 or subpopulation 2 until instant 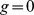. We will refer to these alleles respectively as *type 1* and *type 2*. Starting at 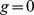, subpopulations will share a common environment for a long period, some genetic mixing will occur, and type 1 and type 2 alleles will not anymore be exclusive of individuals in those subpopulations.

Starting with the initial condition just stated, our model can be fully simulated at the computer. At each generation, the model consists of the following three stochastic steps:

1. *Subpopulation size assignment*

We assume that the total population size is constant and equal to 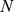. This is motivated by the fact that ecological factors determine the number of individuals that may live in a given region. Nevertheless, the sizes 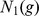 and 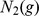 of the two subpopulations at generation 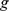, constrained by 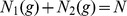, may vary. More exactly, we assume that 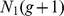 and 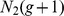 are random variables modelled by the following stochastic rule: any of the 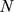 individuals of generation 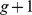 will independently belong to subpopulation 1 with probability 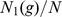 or to subpopulation 2 with probability 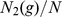.

The above described stochastic process permitting fluctuation of the subpopulation sizes is exactly the same as in the well-known *neutral* Wright-Fisher model for two alleles at a single locus, see e.g. pages 75–84 in [21] or pages 199–202 in [22], with the two alleles traded here for the two subpopulations. The Wright-Fisher model, in its original context, describes the random genetic drift phenomenon. Due to finiteness of 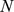, under such a model the numbers 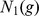 and 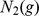 of alleles of either type in a fixed size population fluctuate as generations pass. The values 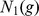, 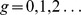 are a realization of a Markov chain with 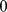 and 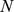 as the only absorbing states. Thus either of the alleles, or returning to our context, subpopulations, will become eventually extinct in a finite number of generations with probability 1. From here on we will refer to the process allowing fluctuation of subpopulation sizes as the *Wright-Fisher process*.

The number of generations until extinction of one subpopulation in the Wright-Fisher process is random, as well as which of the two subpopulations becomes extinct. If 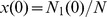 is the initial fraction of individuals of subpopulation 1, it may be shown that subpopulation 1 will survive with probability 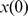. The mean number of generations until extinction is approximately given, for large 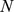, by 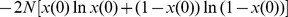 (see [21]). As the mean number of generations for extinction of one subpopulation scales with 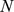, it will be useful to measure time not in generation units, but in generations divided by 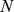. From here on, we will refer to 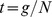 simply as the *time* related to generation 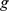.

At any time 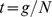, let 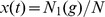 be the fraction of the total population at subpopulation 1. The set of all values 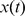 will be called a *history* of the population size.

1. *Migrations*

We assume that at each generation a number 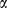 of randomly extracted individuals from subpopulation 1 migrates to subpopulation 2 and vice-versa the same number of random individuals from subpopulation 2 migrates to subpopulation 1. In other words, 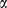 *pairs* of individuals per generation are exchanged, passing from one subpopulation to the other.

As will be seen ahead, the typical values of 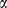 which we will consider are much smaller than 1. In such cases, while simulating the model in the computer, we will produce an event of migration of one pair of individuals each 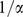 generations, so that 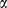 may be interpreted again as the number of pairs of exchanged individuals per generation.

1. *Sexual reproduction*

Remind that the sizes 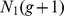 and 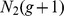 of both subpopulations are already stochastically determined. We proceed by performing 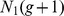 independent random choices of males in subpopulation 1 at generation 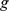. These will be the fathers of the individuals in subpopulation 1 at generation 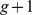. Analogously, we perform independent random choices for the mothers of the same individuals. The first half among individuals in subpopulation 1 will be chosen to be males and the second half will be females. Fathers and mothers of individuals in subpopulation 2 and sex assignment to them are performed analogously.

In this process, individuals which came to some subpopulation at generation 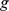 as a result of an exchange process with the other subpopulation will become members of the subpopulation receiving them. In the sexual reproduction process the exchanged individuals will contribute with their genes for generation 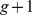 just like any other individual in that subpopulation. Their offspring, if any, is considered as members of the host subpopulation of their parents.

At any time 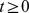 any individual in the total population will be characterized by his/her fractions of type 1 alleles. By our isolation assumption, at 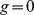 all individuals of subpopulation 1 have fraction equal to 1 of type 1 alleles and all individuals of subpopulation 2 have fraction 0 of such alleles. At further generations, the fraction of type 1 alleles in an individual is the average of its parents’ fractions. We define then 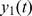 as the *mean fraction of type 1 alleles in subpopulation 1 at time* 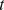 and 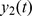 *as the mean fraction of type 1 alleles in subpopulation 2 at time* 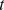. The *mean* here is due to the fact that each individual in any subpopulation in general has different allelic fractions, but 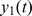 is calculated by averaging the type 1 allelic fractions among all individuals in subpopulation 1, and similarly 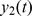 is obtained by averaging type 1 allelic fractions among subpopulation 2 individuals.

The model, as formulated up to now, is fully stochastic and can be simulated in the computer. Although simple, such a simulation for a large population and for a large number of generations is very expensive in terms of computer time. We have produced some small computer simulations of the model for the sake of comparison with better results, which we obtain as we now describe.

It is possible to derive equations relating the mean allelic fractions at generation 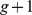 with the mean allelic fractions at generation 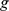. In doing so we will make the assumption that the 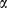 individuals of subpopulation 1 migrating to subpopulation 2 all have an allelic fraction equal to 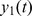. The analogous assumption will be made for all the individuals of subpopulation 2 migrating to subpopulation 1.

Of course the above assumption of exchanged individuals all having the mean allelic fractions in their subpopulations is a very strong one and it is not strictly true. Nonetheless, it is indeed a very good *approximation* if 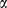 is much smaller than 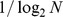. In fact, 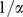 is the mean number of generations between two consecutive exchanges of individuals. As the typical number of generations for genetic homogenization in a population of 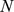 individuals with diploid reproduction and random mating is 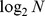, see [23]–[26], the condition that 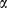 is much smaller than 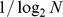 makes sure that subpopulations 1 and 2 are both rather homogeneous at the exchange times. Following nomenclature for a similar approximation common in Statistical Mechanics, the above will be referred to as the *mean field approximation*.

In the mean field approximation, the mean allelic fraction 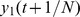 will be equal to 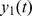 plus the contribution of type 1 alleles from the immigrating individuals of subpopulation 2 and minus the loss of type 1 alleles due to emigration. We remind that these loss and gain terms are both proportional to 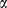 and inversely proportional to the number 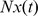 of individuals in subpopulation 1. Similar considerations apply to 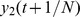. In symbols:
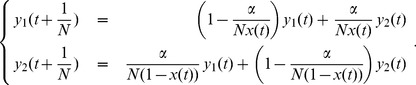
(1)


The above equations, after taking the 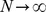 limit, become a system of linear ordinary differential equations (ODEs)
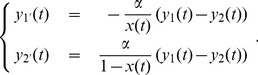
(2)


The above equations describe deterministic gene flow between two subpopulations with stochastically varying sizes, total population size being constant. Although the interbreeding model is fully stochastic, its description through Eqs. (2) maintains only partially the stochastic character, as a consequence of the mean field approximation. Below we will compare the outcome of Eqs. (2) with simulations of the fully stochastic version of the model, showing that the mean field approximation is indeed a good one.

We stress here that we think of 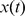 as a Wright-Fisher path, i.e. a a stochastic function obtained by realizing the Wright-Fisher process, but (1) and (2) still hold if 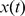 is any description of the history of the size of subpopulation 1, be it stochastic or deterministic. For example, in another work [27] we have explored the possibility of individuals in subpopulation 1 being fitter than individuals in subpopulation 2. As shown there, it is possible to study this situation by using (1) and, in that case, considering the history 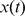 to be deterministic turns out to be a good approximation.

Most of the results we will exhibit are based on performing a large number of times only step 1 in our three-step description of the model. For each Wright-Fisher path 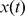 we may numerically solve (2). As numerical solutions of (2) are much easier to compute than the whole simulation of the stochastic migration and reproduction steps, we will be able to obtain a much better statistics than we would have obtained by simulating all the three steps in the model.

### The qualitative behaviour of solutions to the model’s equations

By introducing the auxiliary functions 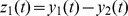 and 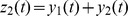 and taking into account the initial conditions 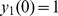, 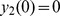, we may solve ODEs (2), obtaining
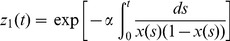
(3)


and
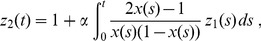
(4)where 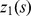 in (4) is given by (3). The same path could be followed for the direct solution of the difference equations (1), but formulae corresponding to (3) and (4) become more complicated. More importantly, the limit 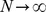 will be appropriate for our further analysis. Of course (3) and (4) may be trivially used to derive explicit expressions for 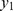 and 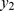, but we think the result is clearer in the form given by (3) and (4).

In general, 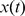 is a complicated function obtained by realizing the Wright-Fisher process. In the 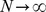 limit, it is a solution of the stochastic ODE
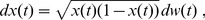
(5)where 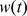 is standard Brownian motion, i.e. 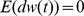 and 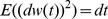. As a consequence, in general we cannot explicitly compute the integrals in (3) and (4). Anyway, (3) and (4) will be used to give a qualitative description of the solutions to (2). For quantitative questions, integrals in (3) and (4) may be easily numerically computed. Equivalently, (1) may be solved by iteration.

As the integrand in the exponent of (3) is positive, this formula shows that 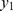 is always larger than 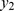, but their difference steadily decreases. Moreover, this information, when plugged into (2) shows that in fact 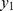 decreases and 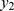 increases. The decay rate of 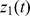 is proportional to the exchange parameter 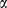 and inversely proportional to the instant value of the product 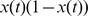. These results mean that the larger 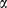 is, the quicker the approach of 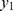 and 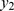 to their final values. Moreover, the approach tends to be slowest when 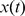 is close to 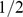 and quickest when either of the two subpopulations is close to extinction.

Eq. (4) on the other hand shows that introgression of genes from one subpopulation into the other is generally not symmetric. In fact, 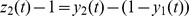 measures the difference between the fraction of type 1 alleles in subpopulation 2 and type 2 alleles in subpopulation 1. By (4), this difference decreases at times in which 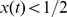 and increases when 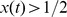. Moreover, it shows that gene flow asymmetries between subpopulations develop more effectively at initial times, when 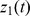 values are larger, and when one of the subpopulations is close to extinction, if 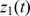 is not too small at that time. The final values of the allelic proportions 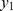 and 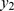, i.e. the values of these quantities at the time one of the subpopulations is extinct, are thus highly sensitive to the history 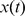, specially its values at times smaller than 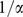. In particular, as extinction times (in generations divided by 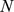) are of order 1, and 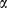 may be small, a large variability will arise in the final values for 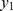 and 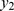. Such a variability can be dealt with by simulating a large sample of histories 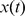, as we will do.

An important special case in which the integrals in (3) and (4) can be exactly evaluated is when 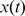 is a constant function, say 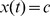. In this case, we get
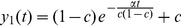
(6)


and
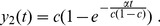
(7)We see that the common value to which both 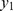 and 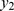 tend in an exponential fashion is just the fixed proportion 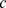 of type 1 individuals in the population.

The importance of this special case is that it provides some useful approximations. For example, if 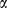 is sufficiently large, then we may consider 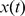 approximately constant during the time of order 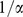 in which the most relevant gene flow between populations will occur. We see that the final values of 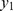 and 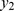 will be close to the initial proportion 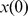 regardless of the behaviour of 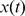 for larger times. Another example is when 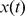 oscillates for a large amount of time around its initial value, a behaviour seen in many realizations of the Wright-Fisher process 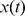. Again, we see that in such a case the final values of 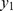 and 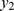 will be close to 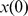.

### Checking accuracy of the model and comparison with its stochastic simulation

**Figure 1:**
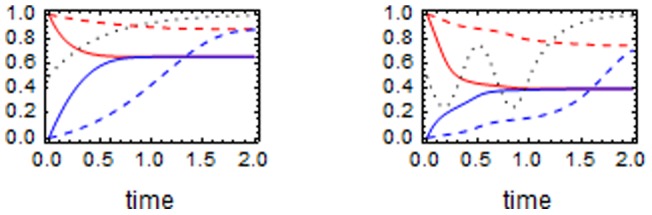
The role of histories 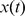 and of the parameter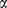. For two different histories 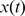 and two different values of 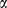 we plot the solutions of (1). In both plots, the black dotted curve represents 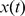. The left plot corresponds to a history in which subpopulation 2 is rapidly extinct, while the right plot to a history in which extinction of population 2 occurs after an initial period in which subpopulation sizes oscillate. In both pictures we represent a situation with 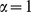 (full lines) and another with 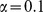 (dashed lines). In each picture the upper (red) lines correspond to 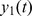 and the lower (blue) lines to 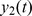. Notice that in these examples the allelic fractions of the subpopulations become nearly equal before extinction.

With the purpose of illustrating the qualitative behaviour of the solutions of (2), we show in Fig. 1 plots of 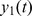 and 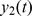 numerically obtained in the case of two deterministic histories 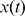 which illustrate typical situations occurring in the Wright-Fisher process.

It can be seen that all qualitative features explained above are present. It should also be noticed that the final values of 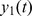 and 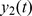, i.e. their values at the time of extinction of one of the subpopulations, do depend very much on the history 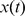 and on the value of 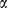.

The final values of 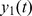 and 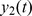 are the most important outputs of the model, because they can be compared with experimental data. As stated above, these values are expected to heavily depend on the particular realization of 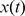 and on 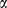. Therefore, although the qualitative behaviour of 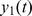 and 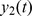 is quite well-understood, it is necessary to numerically solve the model in order to obtain quantitative information on their final values.

**Figure 2:**
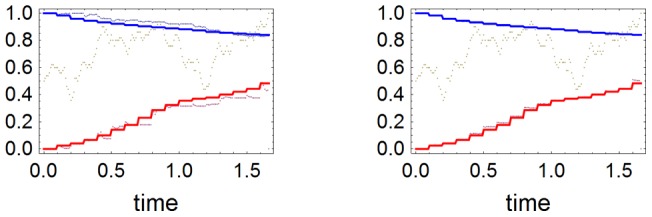
Comparison between theoretical and simulated values for 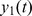 and 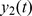. For a single Wright-Fisher path 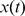 plotted in brown dots and for 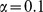 we compare the theoretical and simulated values of 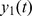 and 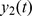. In both plots, the theoretical values are shown in full lines. The upper (blue) line corresponds to 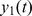 and the lower (red) line corresponds to 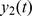.The corresponding simulated values are shown respectively as blue and red dots. The left graph shows the simulated values obtained by a single simulation, whereas the right graph shows the averages of 100 simulations.

In Fig. 2 we check the accuracy of the the approximations leading to Eqs. (2). This is done first by simulating a single Wright-Fisher path 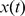, the first step in the model description. In order to do that, we must choose a finite value for 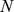. That choice is not so relevant if it is large enough so that agreement between the solutions of (1) and (2) is good. In all results shown we have taken 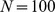, which produced a good agreement.

If we complete simulation of the model by performing the last two steps in the model description, we will obtain what we call the the *simulated values* of the allelic fractions 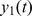 and 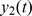. In simulating the sexual reproduction and migration steps, we will also keep track, for each male individual at each generation, of his ancestor by paternal line in 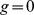. Analogously, for the female individuals we will keep track of the maternal ancestors in 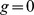. These paternal and maternal ancestors will be used later in showing that our model is compatible with experimental data showing that all living humans are descendants of a single African man for their Y chromosome and of a single African woman for their mtDNA.

On the other hand, we may use the same realization of 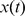 used in the complete simulations, plug it into Eqs. (2) and numerically solve them. The outputs of this are what we call the *theoretical values* of the allelic fractions 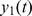 and 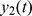.

The left graph in Fig. 2 shows the result of one complete simulation of the model for a population. We obtain the simulated values for 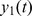 and 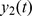 and in the same figure we compare them with the theoretical values using the same Wright-Fisher path 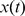. It should be noted that, although not complete, agreement between simulated and theoretical quantities is good. We remind here that the simulated allelic fractions are subject to statistical fluctuations due to the random processes of migration and sexual reproduction.

Indeed, we believe that the randomness in the sexual reproduction process accounts for the largest part of the difference between theoretical and simulated values. In fact, as shown in [23]–[25], with sexual reproduction the contribution of each single individual to the gene pool some generations later is highly variable.

On the other hand, if 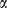 is much less than 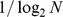, randomness in the process of migration,due to differences in allelic fractions among individuals in the same subpopulation, is not so important, since at the time of exchanges the individuals in each subpopulation are already highly homogeneous from the point of view of allelic fractions. We have directly checked homogeneity among individuals in a subpopulation in the data used to produce Fig. 2. We can safely say that results in Fig. 2 justify the soundness of the mean field approximation.

It should also be noticed that agreement between theoretical and simulated values is worse for 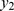 when subpopulation 2 is close to extinction. In this case, in fact, given the small size of subpopulation 2, even a small number of migrants and their variable reproductive success induces large fluctuations in 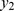.

The right graph in Fig. 2 shows the average of the simulated values 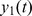 and 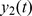 over 100 simulations with the same history. Notice that the difference between theoretical and average simulated values is accordingly smaller.

**Figure 3:**
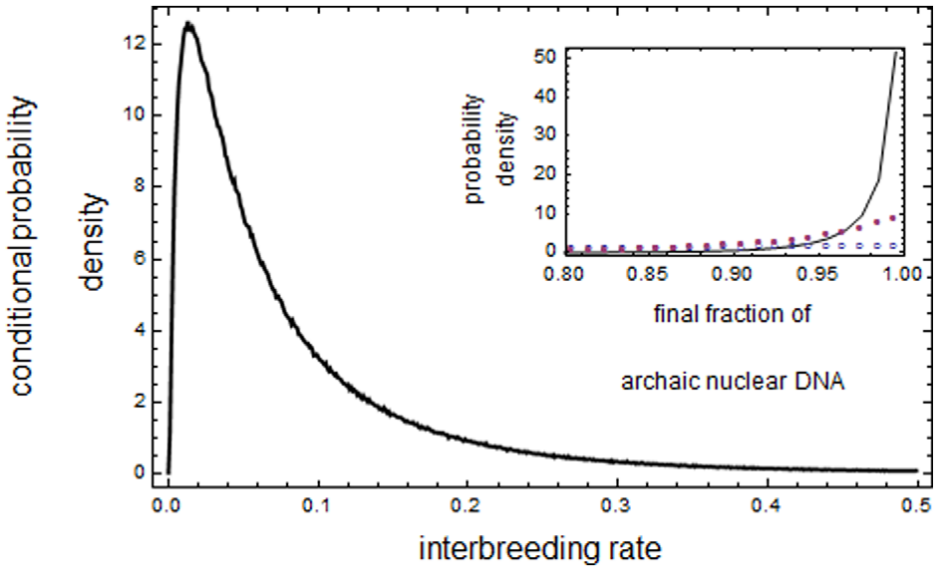
The probability density for 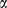. We show here the probability density that the final value of 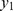 is in the experimental interval 0.96–0.99 as a function of 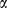. The plot was built by obtaining one million “successful” pairs 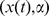 such that subpopulation 2 is extinct and the final value of 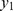 – obtained by solving (1) – lies in the experimental interval. These pairs were obtained out of a total of around 140 million simulated Wright-Fisher paths 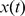 with random 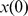 uniformly distributed between 0 and 0.8 and 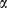 uniformly distributed between 0 and 2. For the successful pairs we then computed the fraction associated to any given 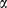. In the inset we plot the probability density for the final values of 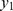 for three different values of 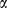. The densities are empirically determined by simulating 400,000 Wright-Fisher paths 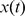 with random 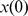 uniformly distributed between 0 and 1 and selecting the histories in which subpopulation 2 is extinct. The empty dots (blue) are data for 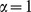, the full dots (purple) are data for 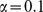 and the full curve (black) are for 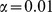.

Although the agreement between theoretical and simulated values is not complete, results shown in Fig. 2 suggest we can trade simulated values by theoretical ones. In fact, only theoretical values have been used in producing e.g. results shown in Fig. 3. In order to produce the data in Fig. 3 we simulated about 140 million Wright-Fisher paths. Such a huge data set could not have been constructed if we had used the much slower simulated values instead of the theoretical ones.

### Estimating the exchange parameter

We know that Neanderthals were extinct and, according to [8], before disappearing they interbred with the African ancestors of Eurasians. Despite comparisons between nuclear DNA of Neanderthals and living humans having been limited in [8] to a sample of only three European Neanderthals and five living humans, the authors of that paper observed that all three Eurasians in their sample are equally closer to the Neanderthals than the two Africans. They estimate that Eurasian living humans possess 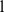 to 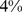 of their nuclear DNA derived from Neanderthals. Identifying the AAE with subpopulation 1 in our model means that the paths 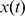 compatible with the experimental data in [8] are the ones in which subpopulation 2 is extinct and the final value of 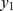 lies between 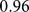 and 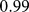. We will refer in the following to the interval between 0.96 and 0.99 as the *experimental interval* for the final value of 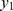.

As we do not know the composition of the total population at the time the two subpopulations met, we will take the initial fraction 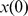 of Africans as a random number. With this hypothesis, after taking the 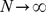 limit, the only parameter of the model to be determined is the exchange rate 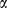.

As can be seen in Fig. 1 the value of 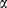 largely influences the final value of 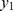. Furthermore, in both Figs. 1 and 2 it can be seen that with 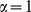 or 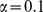 the final values of 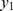 tend to be too small to be compatible with the experimental interval. We stress that these figures are based only on a few realizations of the history 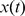 and a single value 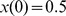. In order to produce estimates of 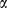 we must produce a large number of histories 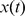 with many values of 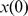 and for any of these simulated histories determine the final value of 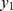 by recursively solving (1).

The inset in Fig. 3 is realized by producing 400,000 Wright-Fisher paths 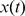 with random 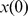 uniformly distributed between 0 and 1. For all these histories we compute the final value of 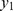 by solving (1) using the three values 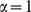, 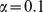 and 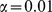. Therefore, for each of the three values of 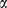 we have about 200,000 data which allow inference of the probability density for the final value of 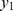. The data plotted in the inset of Fig. 3 show that for 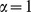 the probability that the final value of 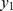 lies in the experimental interval is approximately equal to 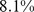. For 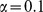 the corresponding probability is approximately of 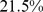 and for 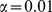 it is approximately of 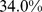. In all three cases the density of the final values of 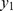 is rather thick, meaning that there is a large probability that the final value of 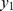 does not lie in the experimental interval.

The above information shows that the experimental data are better explained by values of 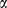 much smaller than 1. By the main plot in Fig. 3 we see that the value of 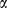 which explains with largest probability the experimental data is 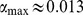. In order to produce that plot, we simulated a large number of Wright-Fisher paths 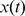 with random 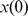 uniformly extracted between 0 and 0.8 and random values for 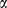 uniformly distributed between 0 and 2. From these data we selected the histories in which subpopulation 2 was extinct and such that the final theoretical value of 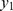 lied in the experimental interval. In this way we can empirically determine the probability that the final value of 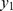 lies in the experimental interval as a function of 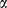.

We also see that the probability density for 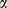 is rather asymmetrical around 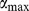, with values 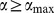 contributing with large probability. This asymmetry is reflected in the fact that the mean value is 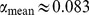, more than 6 times larger than 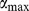.

A technical detail in producing Fig. 3 is that the random values for 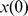 are chosen with uniform distribution in the interval 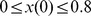, avoiding values between 0.8 and 1, either close to or inside the experimental interval. Such a choice is related to the assumption of *slow* rather than *rapid* interbreeding between Africans and Neanderthals and we now explain why we did so.

First we observe that if 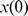 is in the experimental interval, then the final values of 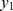 and 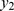 will necessarily also lie in the experimental interval provided that 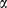 is large enough. This can be proved by Eqs. (6) and (7) and the remark following them.

The free mating situation, in which subpopulations interact as if there were no differences among their members, is a particular case of this large 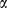 regime. Free mating, in the infinite population limit, is in fact ``described" by (1) with 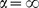. In this case the solution to the equations is straightforward: both 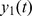 and 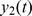 become instantaneously equal to 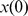.

The conclusion is that if 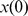 lies in the experimental interval, then the model would fail to predict any upper bound to 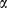, as both quick mating or free mating situations are allowed. Also, we do not believe that either of these situations were likely to have occurred in reality, since distinct subpopulations coexisted for thousands of years. Therefore, the experimental interval has to be excluded in the choice of 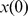.

**Figure 4:**
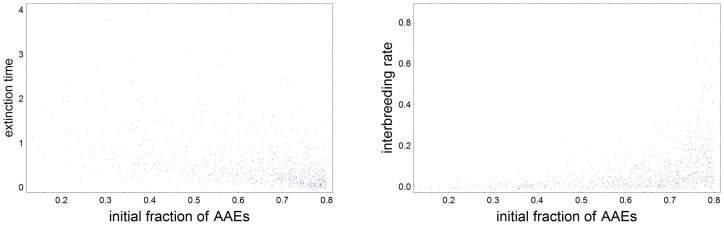
Correlations between 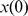 and extinction time and between 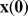 and 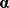. We have produced a large set of Wright-Fisher paths with random 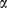 and random 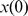 subject to 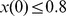. From this set we selected a sample of the 790 histories in which subpopulation 2 was extinct and such that the final value of 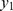 lied in the experimental interval. Both plots in this figure refer to this sample. In the left we show the correlation between 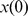 and the time (in generations divided by 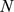) for Neanderthal extinction. The mean extinction time in the sample is 0.58. In the right we plot the correlation between 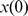 and 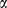. Notice that the number of histories with 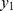 in the experimental interval increases with 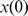, and that larger values of 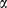 are correlated with large values of 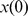.

If we take instead values of 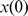 outside the experimental interval, but still close to its boundaries, simulations show that both 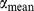 and 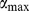 take very large values, such values tending to infinity as 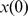 gets closer to the experimental interval. This is illustrated in the right plot in Fig. 4. With 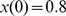 typical values of 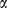 become comparable to 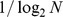 (either with the value 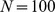 we used for producing that figure and also with 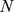 of the order of tens of thousands as it could have been in the real events in Middle East) or larger. As already commented, for such large values of 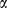, (1) or (2) do not describe accurately the interbreeding process. The reason is that the derivation of Eqs. (1) relies on the mean field approximation, which fails when 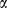 becomes comparable to 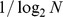.

The right plot in Fig. 4 shows that if we had chosen a value smaller than 0.8 for the right boundary of the same interval, the values of 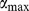 and 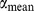 would have decreased. If we had chosen instead a value larger than 0.8, we would incur in too many events with large values of 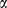, thus threatening validity of Eqs. (1). The choice of the interval 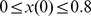, although arbitrary, seems a good compromise between maximality of the interval and soundness of the mean field approximation.

### Mitochondrial DNA and Y chromosome

Mitochondrial DNA and Y chromosome are both inherited in a haploid way. The former is inherited by maternal line and the latter is exclusive to male individuals and, thus, inherited by paternal line. Furthermore mtDNA is not subject to recombination and recombination seems to be negligible for the Y chromosome. It is also believed that large portions of both are selectively neutral. These facts allow an easier mathematical treatment of their statistical properties. From the experimental point of view, mtDNA [1] and Y chromosomes [28] have been sequenced in large samples of living humans. The small variation of both among living humans is compatible with all humans being descendants of a single woman for mtDNA and all men being descendants of a single man for Y chromosome. Further analysis has shown that both the common ancestor for mtDNA and the common ancestor for Y chromosome are probably of African origin and have lived about 100–200 thousand years ago. These facts have been interpreted as proofs of the Out of Africa model.

More recently [29], the whole mtDNA of a few Neanderthal fossils became available. The average number of pairwise differences in mtDNA between a Neanderthal and a living human is significantly larger than the average number of pairwise differences in mtDNA among living humans. This has been considered as a further confirmation of the claims that Neanderthals belong to a separate species, see e.g. [30], and, as a consequence, also for the Out of Africa model, at least with regard to Neanderthal substitution by modern humans.

Both authors of this paper have separately claimed that the above evidences favouring the Out of Africa model are in fact compatible with anatomically modern Africans and Neanderthals being part of a single interbreeding population at the times they coexisted. In [12], using Kingman’s coalescence, it was shown that the probability distribution of genealogical distances in a population of fixed size and haploid reproduction is random even in the limit when the population size is infinite. The random distribution typically allows large genealogical distances among subpopulations. In [13] another important fact was statistically described: in a population of fixed size and haploid reproduction one of the two main subpopulations will become extinct at random times with exponential distribution. Sudden drops in the average genealogical distances among individuals happen at the times of such extinctions. Finally, in [14], it was shown that mtDNA may be completely replaced in a population by the mtDNA of another neighbour population, whereas some finite fraction of its nuclear DNA persists.

These facts imply that the large genealogical distances between living humans and Neanderthals, as seen in mtDNA, are not uncommon in an interbreeding population. On the contrary, they turn out to be very likely if the correct statistics is used. Furthermore, typical distances between individuals in the population formed by Neanderthals and anatomically modern Africans may have been much larger at the time of Neanderthals’ extinction than they are nowadays. They also imply that extinction of Neanderthals’ mtDNA is compatible with the survival of their nuclear DNA.

Exactly the same reasoning can be applied to the mitochondrial and nuclear DNAs of the fossil bones found in Siberia [18], [31], later described as the new population of Denisovans. The fact that Denisovans differ significantly both from Neanderthals and living humans in their mtDNA [31] does not imply that they could not interbreed with either of them. Indeed, nuclear DNA proved [18] that they have interbred at least with some anatomically modern populations.

In [15], [16] the question of survival of mtDNA and Y chromosome lineages in a population subject to exponential stochastic growth (supercritical Galton-Watson branching process) was examined. It was shown that exponential growth is compatible with the survival of a single mtDNA or Y chromosome lineage only if the growth rate is in a narrow supercritical interval. Thus, even if Neanderthals and anatomically modern Africans belonged to the same interbreeding population and even if this population was allowed to grow exponentially with a small rate, the more probable outcome would still be all humans being descendants either of a single woman (mtDNA) or a single man (Y chromosome).

In [17], the number of generations between successive branching events in the Galton-Watson process was computed. It was found that in the slightly supercritical regime, in which the survival of a single lineage is expected, genealogical trees typically have very long branches of the size of the whole tree along with shorter branches of all sizes. Thus, trees are qualitatively similar to those of the coalescent model and, as a consequence, the phenomenon of sudden drops in genealogical distances, described in [13], is also present in the slightly supercritical regime of a Galton-Watson population model.

As explained before, instead of simulating only Wright-Fisher paths (step 1 in the model description), as we did in the results of Figs. 3 and 4, we may simulate also the whole process of reproduction and migration (steps 2 and 3). We ran several simulations of the three-step process, taking populations of 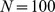 individuals and random values of 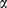 uniformly distributed between 0.01 and 0.2 and random 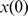 constrained to be smaller than 0.8. Each simulation was run for a number of generations large enough until all male individuals had the same paternal ancestor at generation 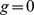 and all female individuals had the same maternal ancestor at generation 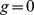. We selected those simulations in which subpopulation 1 survived and 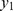 lied in the experimental interval. Out of 96 simulations satisfying the above criteria, only in 7 of them the paternal ancestor of all males and the maternal ancestor of all females were not both of individuals belonging to subpopulation 1 at 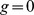. Therefore, according to our interbreeding model, the conditional probability of an African origin for both mtDNA and Y chromosome can be empirically estimated to be of order 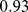. Interbreeding did occur and it shows up in nuclear DNA, but it is perfectly compatible with both mtDNA and Y chromosome for all living humans being of African origin.

### Sizes of interbreeding subpopulations and interbreeding time

Bar-Yosef [10] compares occupation of the Middle East by Neanderthals and Africans with a long football game. The caves of Skuhl and Kafzeh in Israel were alternatingly occupied by anatomically modern Africans and Neanderthals several times over a period of more than 130,000 years. Although the model, as described by Eqs. (2), becomes independent of the total population 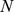, we may obtain some hints on the size of 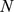 if we accept the constraint that at least for 130,000 years Neanderthals had not been extinct in the Middle East.

By taking random values for 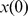 between 0 and 0.8 and 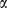 between 0 and 2 we obtained a sample of 790 Wright-Fisher paths such that the MEN subpopulation was extinct and the theoretical final value of 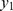 lied in the experimental interval. For each of these histories we recorded the time (in generations divided by 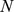) for MEN’s extinction and we found out that the mean extinction time in the sample was 0.58. If we take this mean value as the typical value, suppose that one generation is 20 years and equate it to 130,000 years, we get 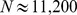 individuals. The whole distribution of extinction times in the sample is shown in the left part in Fig. 4.

In the right part of Fig. 4 we plotted the same sample of events in the plane 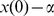. We see that smaller values of 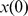 are correlated with smaller values of 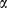 and also that the events such that 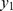 lies in the experimental interval are concentrated around the largest values of 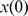. The mean value of 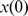 for the whole sample is 0.64. This shows that the small proportion of Neanderthal alleles in living Eurasians, even supposing equal fitnesses for AAE and MEN, does not mean that MEN were much less than AAE in the interbreeding process among these subpopulations.

### Asymmetry of introgression

Our model assumes, for simplicity sake, symmetry in the number of individuals migrating from one subpopulation to the other at each generation. Of course, this does not imply symmetry in the gene introgression of one subpopulation into the other. The reason is that, neutrality assumed, a single individual may change radically the gene pool of a small subpopulation, whereas a single individual in a large subpopulation will probably not alter too much the gene pool of that subpopulation.

**Figure 5:**
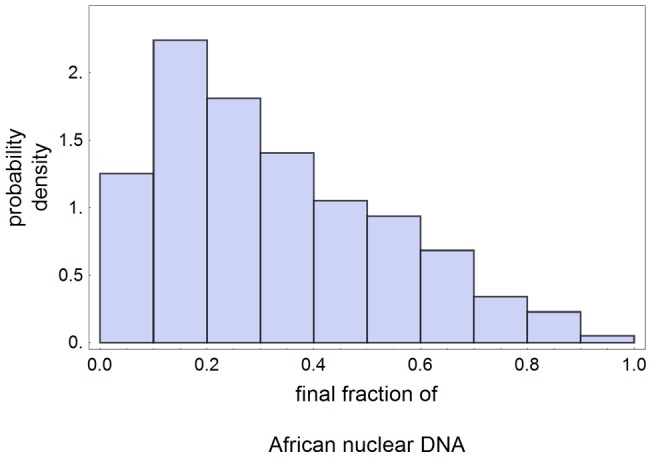
Introgression of African DNA into MEN. We use the same sample of 790 histories used in Fig. 4 for obtaining the probability density for the final value of 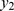 conditioned to MEN extinction and 1 to 4% Neanderthal DNA in AAE. The mean final value of 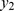 in the sample is 0.33.

This effect is quantified in (4) and explained just after this equation. Moreover, it is clearly visible in Fig. 2. A consequence is that introgression of African alleles into MEN, measured by the final value of 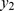, should be large in the histories 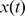 such that MEN are extinct. This is confirmed in Fig. 5, in which we plot the probability density distribution of the final value of 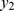 for the same sample of 790 events used in Fig. 4.

On the other hand, Green *et* *al* [8] do not find any evidence of human introgression into Neanderthals. This apparent contradiction can be explained if we suppose that only a part of the total Neanderthal population took part in the interbreeding process in Middle East, the other part of the population remaining in Europe. As the few Neanderthal specimens which had their DNA considered in [8] were all excavated in European sites, it is possible that they had never had contact with Africans.

## Discussion

In the framework of the model proposed in this article we could infer that the 1 to 4% fraction [8] of Neanderthal DNA in present day non-Africans can be explained with maximum probability by assuming that the AAE and MEN subpopulations exchanged only 1 pair of individuals in about 77 generations. The mean value of the exchange parameter in the model corresponds to a much larger frequency of 1 pair of individuals exchanged in about 12 generations.

We also estimated the mean number of generations for Neanderthal extinction in the Middle East to be approximately 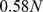, where 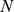 stands for the total interbreeding population. Together with the fact that Neanderthals and Africans seem to have coexisted in the Middle East for at least 130,000 years, this allows us to estimate 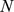 to be of order 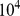 individuals.

Moreover, our model is compatible with the lack of introgression of Neanderthals into mtDNA and Y chromosome of living humans. We estimated in only 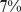 the probability that humans could have either mtDNA or Y chromosome of a Neanderthal origin.

Neanderthals are implicitly considered in this work as a group within the *Homo sapiens* species and we renounce the strict Out of Africa model for the origin of our species, in which anatomically modern Africans would have replaced without gene flow other hominids in Eurasia. In particular, our model is neutral in the sense that we assign the same fitness to Neanderthals and Africans. Our results show that neither strong sexual isolation between Africans and Neanderthals, or else some kind of Neanderthal cognitive or reproductive inferiority, are necessary to explain both their extinction and the small fraction of their DNA in most living humans. In fact, within the assumptions of the model, if two equally fit subpopulations coexist in the same territory for a sufficiently long time, only one of them survives. The fact that Neanderthals were the extinct subpopulation is then a random event.

Although we do not intend to back up any kind of superiority for Neanderthals, our neutrality hypothesis is at least supported by recent results [32], [33] by J. Zilhão *et* *al*, which claim that Neanderthals in Europe already made use of symbolic thinking before Africans arrived there.

In another work [27] one of us studied a similar model for interbreeding of Neanderthals and Africans in the Middle East, but trading the neutrality assumption for consideration of various values for the fitness difference of Africans over Neanderthals. The analysis is based still in Eqs. (1), but some technical differences arise. One of the main conclusions is that even with fitness differences as small as 1%, Neanderthals’ extinction comes up in too short a time. The neutral model of this paper looks thus more suitable for explaining a long period of coexistence of Africans and Neanderthals in the Middle East.

In building the model presented here, one of the strongest concerns of the authors was simplicity. As a consequence of this concern, our model contains a single parameter to be estimated, Eqs. (2) are exactly solvable, and qualitative properties of their solutions (3) and (4) are well understood. Computer simulation was only moderately employed. The use of the Wright-Fisher process as a model for extinction under neutrality of one subpopulation seemed to us more natural than if we had assumed some arbitrary population model with other parameters.

After a first version of this work had been published on line at arxiv.org and reviewed by New Scientist [34] on April 2011, Currat and Excoffier published their paper [11] dealing with the admixture of Neanderthals and humans. Besides several other differences between our works, both in methods and in assumptions, we stress here that the model by Currat and Excoffier is more detailed, more complicated and with many more parameters. As a consequence, their results can only be obtained - with less intuitive understanding - by heavy computer simulation. Despite that, both works agree in the conclusion that the number of successful interbreeding events leading to the 1 to 4% introgression of Neanderthals into Eurasians should be small.

Whereas we think of neutrality and no biological barriers, with social barriers preventing free mating, they consider a model of African range expansion, free mating with Neanderthals, but strong reproductive isolation between the “ `two species”, probably due to avoidance of interspecific matings, a low fitness of hybrids, or both. They consider several scenarios for the location of Neanderthals during interbreeding, including the possibility of interbreeding restricted to the Middle East, as we did. Nonetheless, the scenario they consider the more probable is the one in which Neanderthals were scattered through a large geographical area including Middle East, Europe and Central Asia. In particular, in the more probable scenario, their model forecasts interbreeding hotspots also in Europe and Central Asia, very far from the Middle East.

We identify two important differences between our assumptions and those of Currat and Excoffier, which could possibly be resolved by future experimental tests. The first difference is that although both models predict asymmetric introgression among interbreeding subpopulations, ours predicts larger introgression of Africans among Middle Eastern Neanderthals. Theirs, on the contrary, predicts smaller introgression of Africans into any Neanderthals. Their prediction is in agreement with the results of Green *et* *al* [8] which saw no introgression of Africans into *European* Neanderthals.

Our prediction also agrees with this fact, if we suppose that a fraction of the Neanderthal population never left Europe and did not participate in the postulated Middle Eastern interbreeding. The descendants of these European Neanderthals did not interbreed later with Africans when they came into Europe, or this interbreeding was very small, possibly due to small population densities of the Neanderthals when they were close to definitive extinction. DNA sequencing of late Middle Eastern Neanderthal fossils and comparison with European Neanderthals would be a good test for helping discriminate between the two models.

The second difference is that our model is simpler in that it does not take into account the spatial distribution of the subpopulations. Along with [8] we suggest that interbreeding occurred only in the Middle East. On the other hand, Currat and Excoffier find it more probable that interbreeding occurred in a large region including Europe and Central Asia. Although we have not addressed the question of whether introgression of Neanderthal alleles was the same for all *loci* or not, it is conceivable that in our model roughly the same Neanderthal alleles will be present among all Eurasians. On the contrary, Currat and Excoffier suggest that different Neanderthal alleles may be present among living Asians and Europeans. In this case, an experimental test with living humans might resolve the controversy.

Current knowledge about Denisovans’ morphology and life style is much less than what we know about Neanderthals. In particular we do not know whether Denisovans lived only in Siberia, where up to now the only known fossils have been found, or elsewhere. Where and when this people made contact with the African ancestors of present day Melanesians and Australians is still a conjecture [19]. Nevertheless, if such a contact occurred for a sufficiently long time in a small geographical region, then the present model can be straightforwardly applied.

As we now know of our Neanderthal and Denisovan inheritances, it is time to ask whether they were the only hominids that Africans mated. We believe that the future may still uncover lots of surprises when Denisovans will be better studied and nuclear DNA of many more Neanderthal and other hominid fossils will become available. In particular, we expect that in a near future experimental tests and archaeological or palaeontological discoveries may discriminate where, when and for how long Africans interbred with Neanderthals and other hominids, and prepare the way for finer theories.
